# Viral metagenomics reveals persistent as well as dietary acquired viruses in Antarctic fur seals

**DOI:** 10.1038/s41598-022-23114-y

**Published:** 2022-10-28

**Authors:** Sandra Martínez-Puchol, Luis Cardona, Massimiliano Drago, Manel Gazo, Sílvia Bofill-Mas

**Affiliations:** 1 Laboratory of Viruses Contaminants of Water and Food, Departament de Genètica, Microbiologia i Estadística, Facultat de Biologia, Universitat de Barcelona (UB), Barcelona, Spain; 2 Institut de Recerca de l’Aigua (IdRA), Universitat de Barcelona (UB), Barcelona, Spain; 3 Departament de Biologia Evolutiva, Ecologia i Ciències Ambientals, Institut de Recerca de la Biodiversitat (IRBio), Facultat de Biologia, Universitat de Barcelona (UB), Barcelona, Spain

**Keywords:** Virology, Metagenomics, Microbial ecology

## Abstract

Viruses linked to animals inhabiting Antarctic latitudes remain poorly studied. Remote environments hosting large pinniped populations may be prone to exposure of immunologically naïve animals to new infectious agents due to increasing human presence or introduction of new animal species. Antarctic fur seals (*Arctocephalus gazella)* inhabiting the Western Antarctic Peninsula and the South Shetland Islands are challenged because of climate change and increased anthropogenic activity. In the present study, the fecal and serum virome of *A. gazella* was characterized by applying target enrichment next generation sequencing. The resulting viromes were dominated by CRESS-DNA sequences. Viruses known to infect vertebrate and invertebrate hosts were also observed in fecal samples. Fur seal picornavirus was present in all the fecal pools studied suggesting it is a prevalent virus in these species. Six different viruses presenting similarities with previously described *A. gazella* viruses or other otariids and mammal viruses were identified as potential new *A. gazella* viruses. Also, diet-derived viruses such as crustacean viruses were present in fecal content. Penguin viruses, but not fish viruses, were also detected. Obtained results contribute to a better understanding of the viral community present in these species, which is relevant for its conservation.

## Introduction

Antarctica is considered the most pristine environment on the planet, due to a very small and mostly seasonal human presence^1^. However, human presence in the region is increasing and the South Shetland Islands and the western Antarctic Peninsula is currently the most affected region^2^. Increased levels of human activity results in increased exposure of wildlife to pollutants such microplastics^3^ and heavy metals^4^ and even enteric bacteria carried by humans^5^. Furthermore, the western Antarctic Peninsula and the Atlantic sector of the Southern Ocean are experiencing accelerated warming^6^ and changes in sea ice extent have been recently linked to the infection of sea otters in the North Pacific Ocean by the Phocine distemper virus in 2004^7^.

The Antarctic fur seal (*Arctocephalus gazella*) is a midsized pinniped with a circumantarctic distribution, although most breeding colonies occur in islands close to the Antarctic Convergence^8^*. A. gazella* are completely recovered from the severe commercial hunting occurred during the nineteenth century when population numbers decreased below 3000 individuals and it was also believed to have lost genetic diversity^9^. Since then, *A. gazella* have increased in numbers (four to seven million individuals), extended its distribution range and are at minor risk of extinction. The species is listed as Least Concern in the IUCN Red List of endangered species and listed in Appendix II of CITES appendices. The population of the *A. gazella* breeding at the South Shetland Islands is of particular interest, because this is the Antarctic region with the highest human presence^1^.

According to Barbosa and coworkers^10^, field researchers handling animals are the human group posing the highest risk of pathogens transmission to animals while tourists and other personnel pose a significant risk only when near Antarctic fauna. Sewage disposal could be also a source by which human pathogens could be introduced into these ecosystems^11^.

While some early research described the infection of *A. gazella* by parasites^12^ and bacteria such as *Camplylobacter*, *Streptococcus spp* or *Staphylococcus schleiferi* subspecies coagulans^13–15^, few studies have attempted to identify viral pathogens that infect these populations. All in all, only a few descriptions of viruses infecting *A. gazella* are available in literature. A novel virus named fur seal picorna-like virus^16^ and two new lineages of anelloviruses^17^ have been the unique descriptions of virus infecting *A. gazella* obtained from a fecal sample and bucal swabs respectively. Antibodies against phocine herpesvirus 1 were detected in 58% of *A. gazella* individuals examined by Tryland et al.^18^ but no other evidence of this infection has been reported since then.

The scats of these animals may contain viruses that infect them and replicate along the digestive system. Also, fecal contents may harbour the viruses that infect the animals ingested by *A. gazella* those infecting any animal that is part of *A. gazella* trophic chain. Additionally, serum samples may present viruses when they use blood as a route to reach their target organs from the site of primary infection, to disseminate from them or as a tissue to infect when blood cells are the target of viral infection.

Here, we evaluated the viral diversity of male *A. gazella* hauling out at Deception Island (South Shetland Islands) by applying next generation sequencing. Feces as well as serum samples were studied for the presence of viruses by applying targeted and untargeted enrichment mass sequencing.

Viral high throughput sequencing in complex samples, as the ones analysed in this study, is challenging since genomes from viruses are in low concentrations compared with other microorganisms or host genome, and consequently their sequencing requires the use of specific protocols to ensure its recovery. Target enrichment protocols, usually based in a probe-based enrichment, proved to be the best methodologies to overcome these drawbacks, enabling the sequencing of only specific viral groups of families^19–21^. In this study, the application of two different sequencing approaches intended to provide different viral information from viruses infecting *A. gazella:* enrichment targeted against viruses infecting vertebrates and untargeted viral metagenomics for the characterization of other viruses related to their feeding which might have invertebrates as a host.

## Results and discussion

After massive parallel sequencing of the nucleic acids obtained from fur seal scats, a wide variety of invertebrate and vertebrate viral hosts assignations with low nucleotidic and amino-acidic identities were obtained, most of them corresponding to animal species not described before in Antarctica. These results make us reconsider the use of closed RefSeq databases for viral discovery, especially because the studied area was a remote geographical area where a high number of new viral species is expected to occur^22^.

After repeating the analysis of the contigs obtained using BLASTn, a high number of miss-assignments was observed, corresponding almost entirely to contigs newly assigned as unclassified Eukaryotic Circular Rep-Encoding Single-Stranded DNA (CRESS-DNA) viral sequences. CRESS viruses have been detected ubiquitously in many different animals without any recognised role in the development of any disease^23–26^.

These results are in accordance with the recent reporting of CRESS sequences also being ubiquitous in a wide variety of environments and at high proportions, including Antarctica, where they have been described to represent more than 50% of sequences obtained from glacier waters^27^.

### Viral-host distribution

Virome studies in other *Arctocephalus* species from subantarctic and South American regions revealed a 5% of viral sequences with predominance of bacteriophages followed by viruses from the *Parvoviridae* family^28^. The methodology here applied provided an increase of 12–25% viral reads when probe-based Target Enrichment Sequencing (TES) was applied, that in comparison with Untargeted Viral Metagenomics (UVM) approaches conducted in these type of samples^28^ could be considered an optimal result.

**Figure 1:**
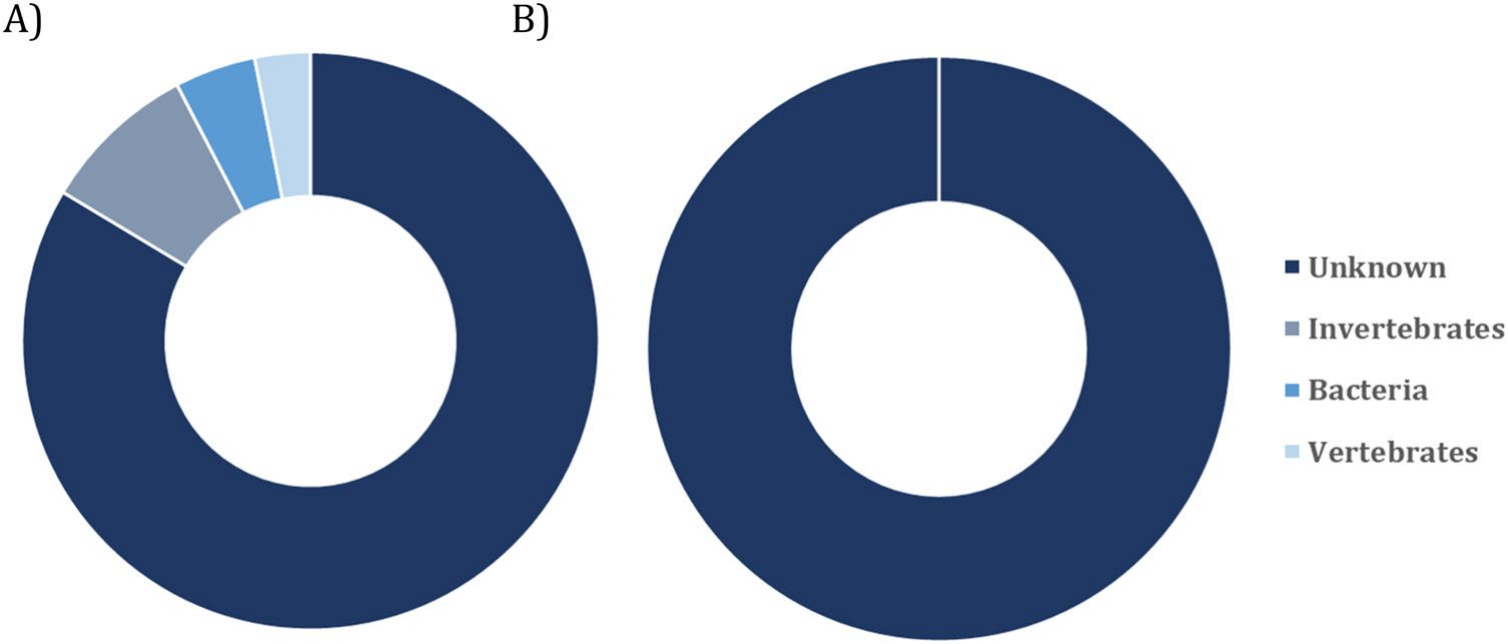
Host distribution of viral assignations sequenced from fecal (**A**) and serum (**B**) samples collected from male *A. gazella*.

Most of the viral species detected in feces corresponded to unknown viruses, 83.59% from the total of sequences, followed by viruses that infect invertebrates, 8.75%, bacteriophages, 4.46%, and vertebrate viruses, 3.11% (Fig. 1).

As expected, when applying both targeted and untargeted sequencing methodologies, TES approach resulted in a recovery of many vertebrate viral assignations (Table 1) whereas untargeted sequencing enabled a better detection of viruses known to infect invertebrates (Table 2). To describe the complete *A. gazella* fecal virome, sequences obtained by both sequencing methodologies were considered all together, representing a total of 2.62 million reads.

**Table 1:** Vertebrate viral assignations obtained from fecal samples sequencing from male *A. gazella*. Ranges of Genome coverage, nucleotide identity and aminoacidic identity are expressed in percentages.

Viral Assignment	Reads	Contigs	Coverage	NT ID	AA ID	Presence in pools (n = 4)	NGS method
Fur seal picorna-like virus	2671	19	19.75–96.91	45.92–99.38	33.23–60.40	4	TES/UVM
Torque teno *A. gazella* virus 2	25	1	3.42	95.12	90.8	1	TES
Mamastrovirus	1008	4	14.01–54.97	45.70–59.37	33.69–46.76	2	TES
California sea lion norovirus	56	1	4.43	99.1	97.5	1	TES
Adeno-associated virus—2	138	3	17.78–22.16	46.91–48.04	35.18–83.12	2	TES

**Table 2:** Invertebrate viral assignations obtained from fecal samples sequencing from male *A. gazella*. Colours represent the presence of each assignation in the processed pools. Ranges of Coverage, NT ID and AA ID are represented in percentages.

Viral assignment	Viral assignment host		Reads	Contigs	Coverage	NT ID	AA ID	Presence in pools (n = 4)	Method of detection
	Classe	Name							
Beihai narna-like virus 19	Annelida	Sipuncula	22	1	81.22	46.6	31.79	1	UVM
Beihai picobirna-like virus 7			28	2	26.08–31.20	44.10–46.40	34.87–41.84	2	UVM
Beihai picorna-like virus 43			77	2	11.16–12.89	48.45–49.08	36.95–43.05	2	UVM
Beihai picorna-like virus 54			5	1	35.75	52.38	62.71	1	UVM
Beihai narna-like virus 9	Arthropoda	Merostomata	26	3	11.84–99.38	42.69–46.76	31.54–47.9	3	UVM
Beihai picorna-like virus 81			4	1	65.07	43.44	55.78	1	UVM
Beihai sobemo-like virus 1	Cephalopoda	Octopus	81	3	13.17–79.21	42.73–76.5	42.07–96.64	3	TES/UVM
Beihai sobemo-like virus 3			146	3	12.14–18.07	47.83–61.17	37.43–80.55	3	TES/UVM
Beihai sobemo-like virus 5			85	2	22.12–99.46	46.35–61.68	34.64–88.50	1	TES/UVM
Wenling crustacean virus 2	Malacostraca	*Sicyonia* sp.	203,514	2	49.27–52.22	98.47–99.36	48.27	1	TES/UVM
Wenling crustacean virus 3			21	2	22.88–72.62	45.87–66.79	34.1–41.09	2	TES/UVM
Wenling tombus-like virus 4			25,308	4	15.43–69.35	45.18–64.57	41.04–76.43	3	TES/UVM
Halhan virus 1	Gastropoda	*Halioti*s sp.	11	2	44.54–45.44	50.64–95.10	48.20–50.64	2	UVM
Wuhan millipede virus 3		Diplopoda	2	1	32.94	52.35	44.08	1	UVM
Sea star-associated densovirus	Asteroidea	Asterozoa	6	1	82.37	42.31	64.22	1	UVM

### *A. gazella* viruses

#### Fur seal picorna-like virus

Fur seal picorna-like virus was firstly described in a fecal sample obtained from *A. gazella* in King George Island in the South Shetland Islands, Antarctica by Krumbholz and co-workers^16^.

**Figure 2:**
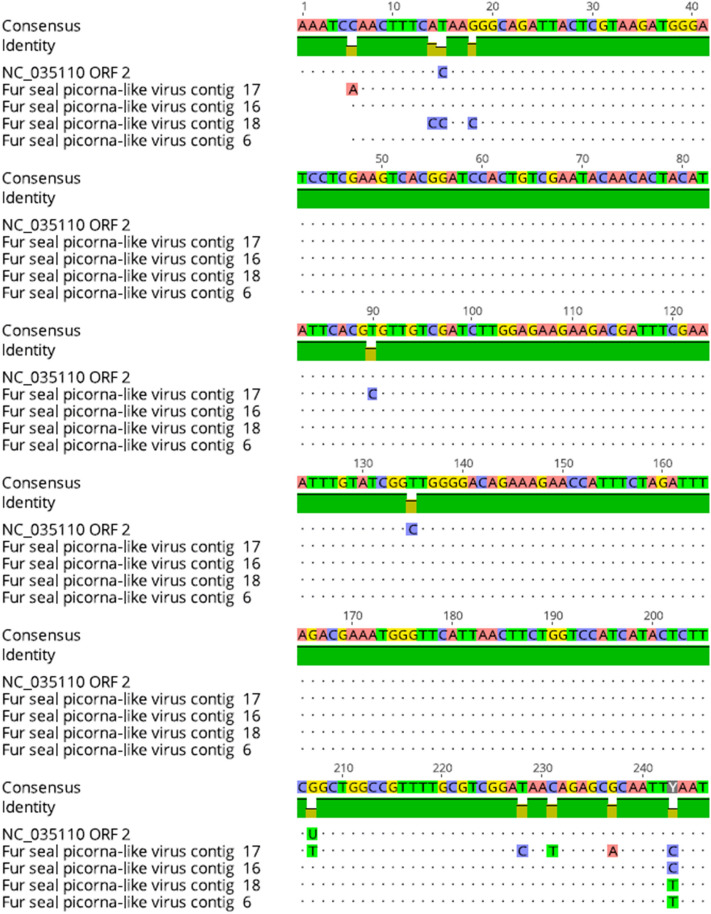
Nucleotide alignment of ORF2 sequences from the *A. gazella* picorna-like contigs compared to the ORF2 from RefSeq NC_0351110. In consensus strain, position 1 represents position 6523 from RefSeqs genome.

In this study, we report a total of 19 contigs resulting after assembling 2671 reads obtained from 4/4 fecal pools analysed being the most prevalent virus described in this study. One of the contigs covered 96.91% of the fur seal picorna-like virus genome and presented a nucleotide homology of 99.38% with the reference strain described in 2017. The other contigs coverage ranged from 19.75 to 21.22% with a 45.92 to 90.5% nucleotide identity with reference strain NC_035110. Four contigs matching the ORF2 polyprotein are represented in Fig. 2 where differences among them and with the reference strain are showed.

Picornaviruses are known to cause a wide variety of diseases in vertebrate hosts, especially mammals^29^, but the role of Fur seal picorna-like virus in pathogenesis development is still unknown^30^. Many picornaviruses are transmitted horizontally via fecal–oral or airborne routes^29^. The fact that these sequences were detected in all the fecal pools obtained from animals with no evidence of disease may that suggest the virus may have a stable endemic relationship within that seal population.

#### Torque teno pinniped virus

*Lambdatorquevirus* is a genus within the *Anelloviridae* family. The genus comprises 8 species named Torque teno pinniped virus 2 to 9 isolated from different pinniped species: *A. gazella* (Torque teno pinniped virus 6 and 7)^17^, *Phoca vitulina* (Torque teno pinniped virus 2, 3, 4)^31^, *Zalophus californianus* (Torque teno pinniped virus 5)^32^ and *Leptonychotes weddellii* (Torque teno pinniped 8 and 9)^33^.

One contig with a nuleotide similarity of 95.12% against Torque teno pinniped virus 7 was obtained from one of the fecal pools. This virus had been described in these animals inhabiting Livingston Island in 2016, using rolling circle amplification and subsequent Sanger sequencing from buccal swabs^17^. However, sequences obtained in this study belong to partial ORF2 which is not the optimal genome region for typing purposes or phylogenetic analysis.

These members of the Anelloviridae represent the more abundant viruses found in human, animals and environmental samples although their etiological role in any disease has not been clearly identified being considered a persistent virus ubiquitous to several different tissues^34,35^

No Torque teno virus sequences were detected in serum samples which agree with what was observed for *Zalophus californianus* anellovirus prevalently detected in different tissues, like lung and liver, but not in blood samples. Interestingly, other known anelloviruses are typically found in blood or plasma samples^32^.

#### Mamastrovirus

**Figure 3:**
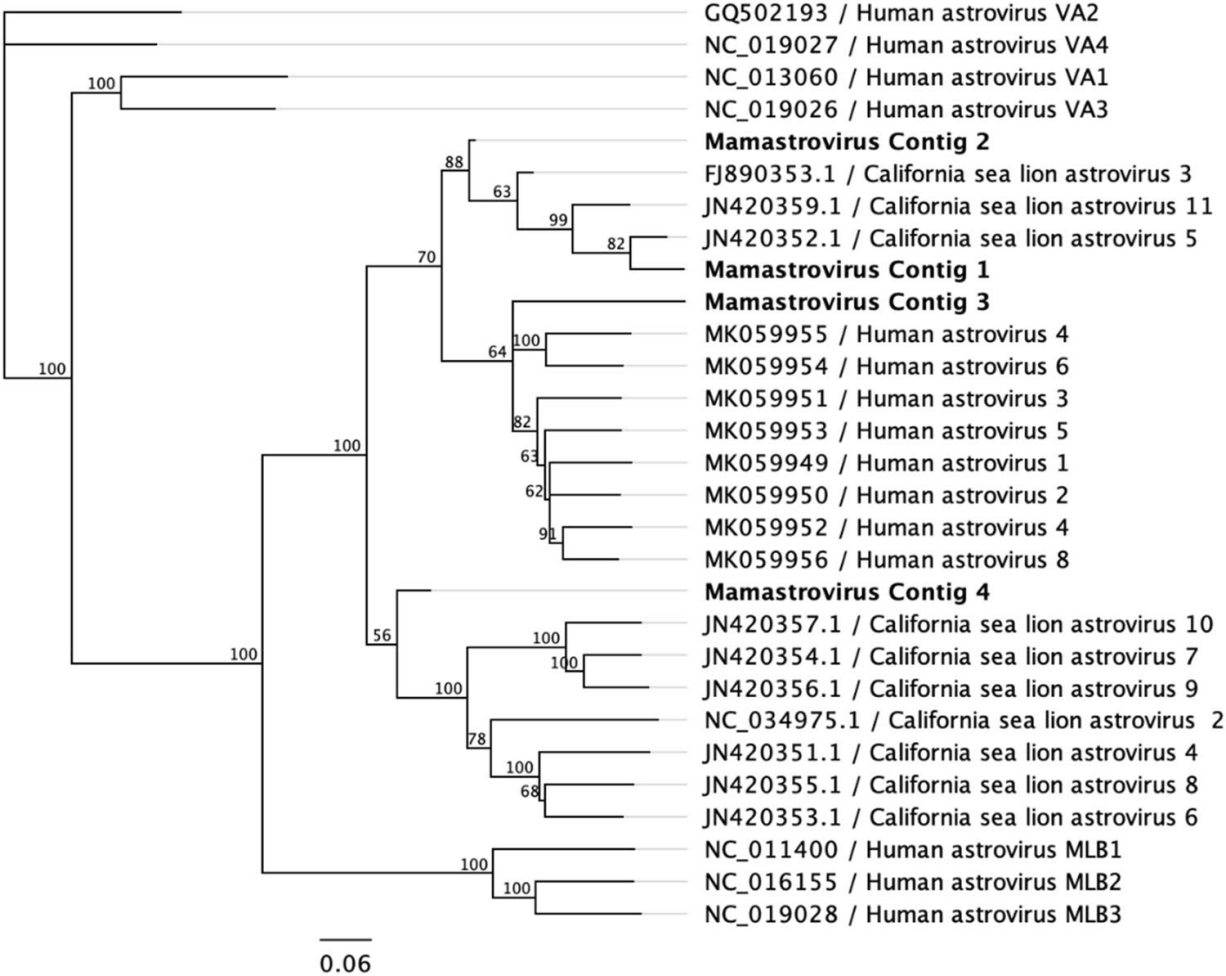
Phylogenetic consensus tree based on partial ORF2 sequences from the Mamastrovirus contigs sequenced from *A. gazella* scats (in bold). Bootstrap resampling with 1000 replicates.

Two of the fecal pools analyzed presented Mamastrovirus sequences. The presence of these viruses in humans and other mammals is widely known, as well as their involvement in gastroenteritis development^36^. The four contigs obtained (comprising 1008 sequences) showed homologies against reference genomes, ranging from 45.70% to 59.37% when compared at nucleotide level and 36.69% to 46.69% when compared at aminoacidic level. Phylogenetic analysis of partial OFR2 regions of these contigs indicate its closer similarity with sequences from California Sea Lion astroviruses, a virus that was determined as to be the most prevalent in fecal samples from these animals (*Z. californianus*)^37^. This finding suggests that these sequences may belong to a yet unknown virus like *Z. californianus* astrovirus and may indicate that such virus is prevalent in the sampled area (detected in 2/4 fecal pools studied) and the second more abundant virus (1008 reads) in the studied fecal samples (Fig. 3).

#### Adeno associated virus 2

**Figure 4:**
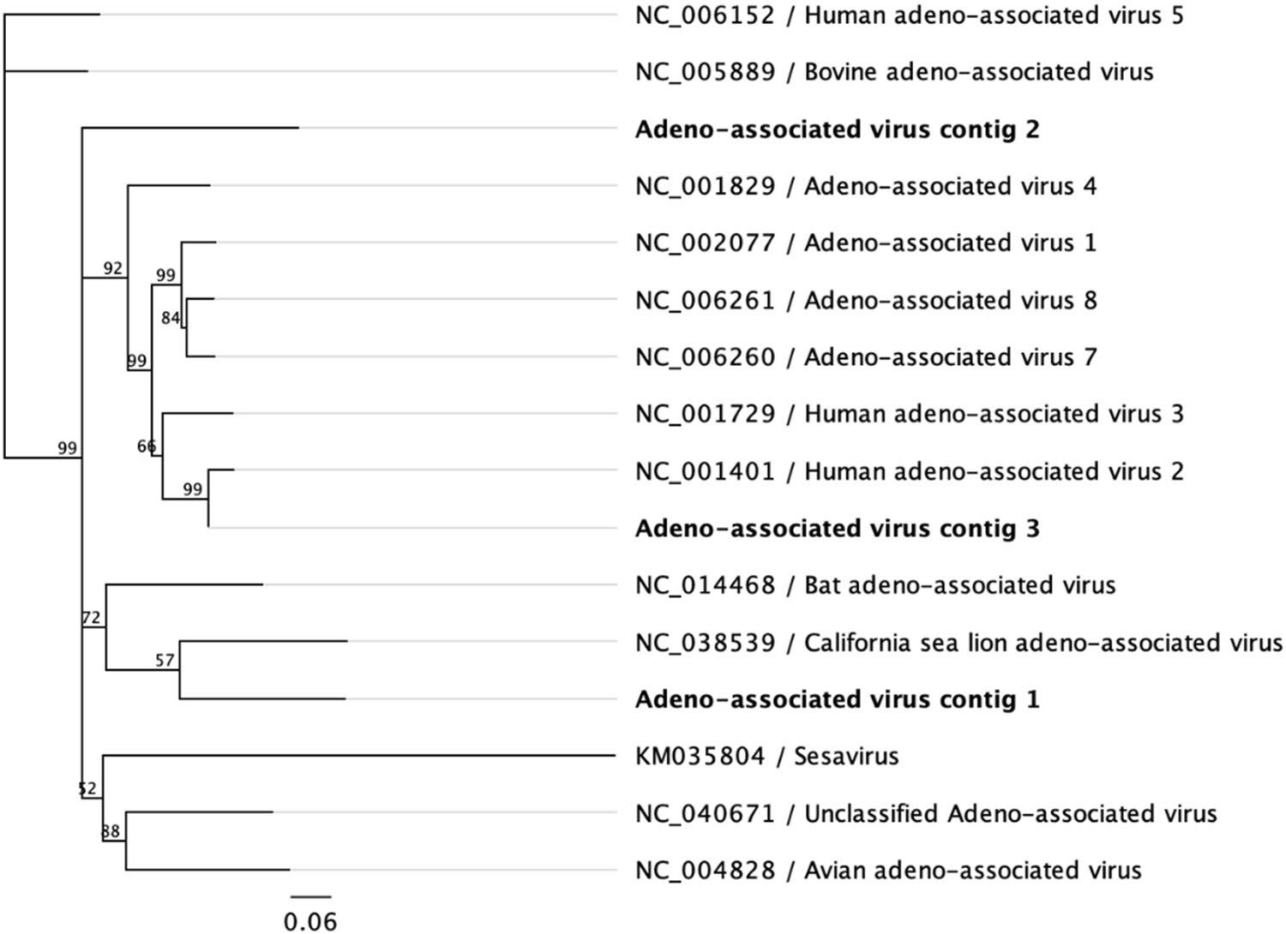
Phylogenetic consensus tree of the Adeno-associated virus contigs sequenced from *A. gazella* scats (in bold). Bootstrap resampling with 1000 replicates.

Two of the studied fecal pools presented 138 sequences, forming 3 contigs with nucleotide identities ranging from 46.91 to 48.04% (Table 1), that matched adeno associated viruses previously described in *Z. californianus*, humans and other mammals with and unknow etiologic role (Fig. 4). The detected sequences probably correspond to fur seal adeno associated viruses never described before. The detection of these viruses is quite common in other mammals suggesting they could cause persistent infections in their hosts, but no etiological role has been attributed to them^38^.

#### Norovirus

**Figure 5:**
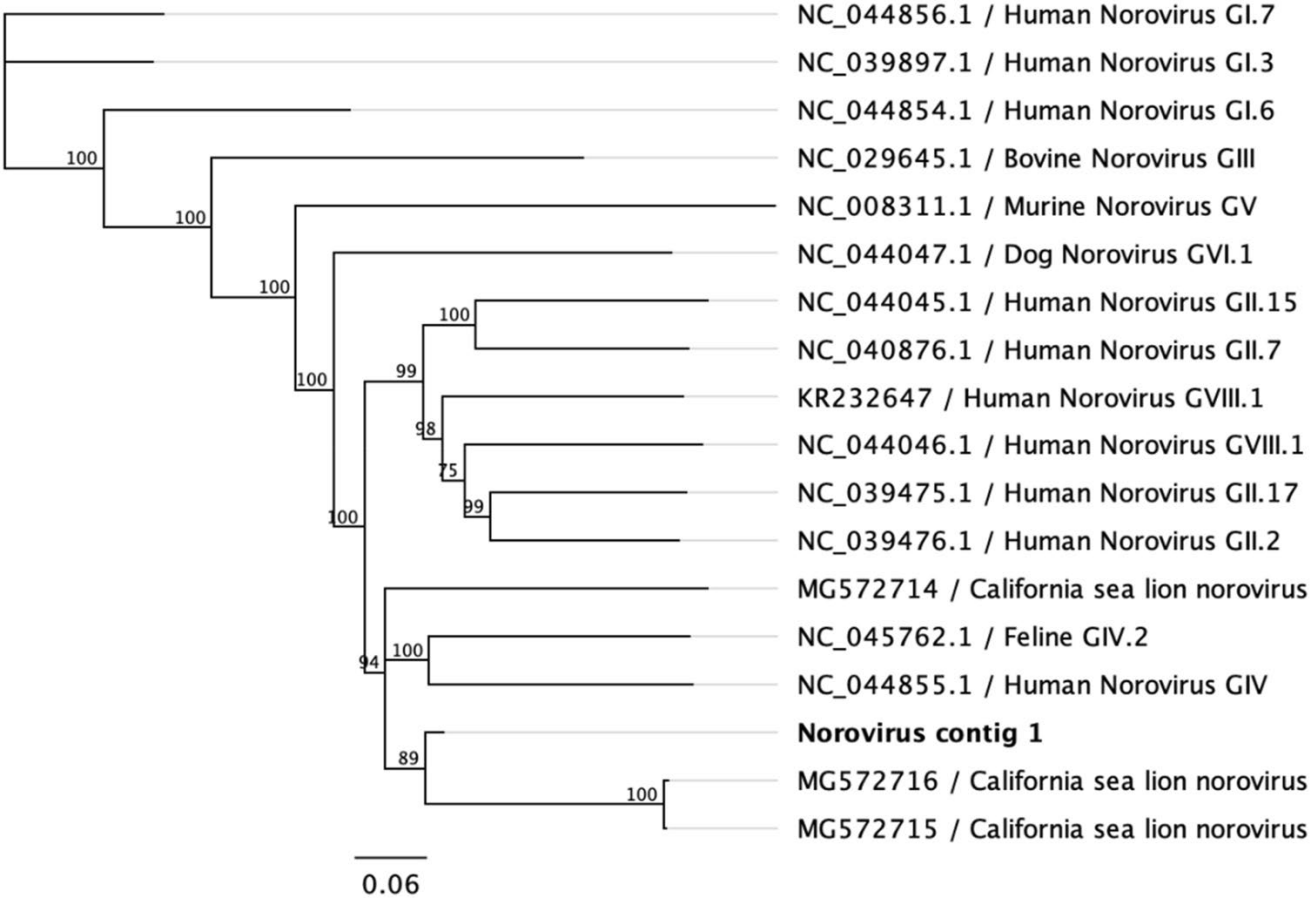
Phylogenetic consensus tree of the Norovirus contig sequenced from *A. gazella* scats (in bold). Bootstrap resampling with 1000 replicates.

A norovirus contig was obtained in one of the four pools analyzed. Noroviruses are the most relevant non-bacterial gastroenteritis etiological agents in humans^39^, with its presence widely described in other mammals^40^. The contig detected in the fecal samples, represented the 4.43% of the viral genome, was in the VP1 region and comprised 56 reads with an identity > 99% to California sea lion norovirus described by Teng and collaborators in 2018^41^ (Fig. 5). Results obtained suggest these sequences belong to a putative new norovirus specie.

### Viruses in serum samples

All the viral sequences obtained from serum samples (970 reads) matched to CRESS-DNA viral sequences from unknown hosts.

The fact that no other viruses were identified in serum samples suggests the animals tested were not under active viremia at the time of sample collection or it was not detectable by the applied methodology.

### Diet related viruses

Several virus sequences similar to viruses known to have invertebrate animals as hosts were detected in fecal pools, mainly by UVM although some also by TES. These viruses are probably present in fur seal feces because of dietary habits although, since scats were collected from the ground nearby the animals, environmental cross-contamination should not be ruled out.

Sequences with high coverage or similarities to any described virus are showed in Table 2.

The high prevalence of virus sequences from crustaceans in the feces analyzed is hardly surprising because *A. gazella* inhabiting the Antarctic peninsula and the Atlantic sector of the Southern Ocean feed mostly on Antarctic krill *Euphasia superba* during the summer months^42–48^. Sequences from cephalopod viruses were also detected, although were much scarcer than those from crustaceans. This also agrees with current knowledge about the diet of *A. gazella* in the Atlantic sector of the Southern Ocean, where octopuses and squids are regularly consumed, although in low numbers^44–46^. It is worth noting than not cephalopod beak was recovered from the scats analyzed here^48^. Among all invertebrate viruses identified, some sequences present low identities with genomes from available databases, probably because Antarctica wildlife has been scarcely explored, forcing bioinformatic analysis to match them with the most similar viruses from these databases.

No fish viruses were found in this study. Hard skeletal remains of fishes are often recovered from the scats of *A. gazella* from the Atlantic sector of the Southern Ocean^42–47^ and occurred indeed in the samples analysed here^48^, but stable isotope analysis of blood and whiskers revealed a negligible contribution of fish to the assimilate diet of juvenile and subadult male *A. gazella*^49^, which likely explain the absence of fish viruses in the samples analized here. Additionaly, no data on the virome present in the fish species regularly consumed by *A. gazella* has been published to our knowledge, with information limited to the bacteriome^32^, so even in case fish viruses were sequenced, it might not be correctly assigned to a fish host. Nevertheless, the methodology applied in this study had been successfully applied to the identification of the virome of Atlantic fishes^50^. Furthermore, Li and coworkers.^37^ and Wille and coworkers.^22^ also observed viral sequences probably corresponding to fish when analyzing the fecal virome of the California sea lions and Antarctic penguins.

On the other hand, sequences highly similar to Coelho and Khabarov viral polymerases (greater than 98% of aminoacid identity), previously described in chinstrap penguins (*Pygoscelis antarcticus*) by Wille and coworkers^22^, were found in this study. The consumption of penguins by *A. gazella* during the summer months has been reported widely^51–55^, penguins feathers were reported from the scats analyzed in this study^48^ and stable isotope analysis of blood and whiskers revealed penguins as the second most relevant prey from juvenile and subadult male *A. gazella* in the population studied here^49^. This evidence is consistent with the presence of virus from chinstrap penguins in the samples analysed here. All in all, the study of fecal virome constitutes a very promising tool to explore the consumers’ diet.

## Conclusions

Targeted enrichment sequencing applied to this study allowed the detection of viruses that had not been detected by untargeted viral metagenomics, highlighting the appropriateness of using these approaches for the study of viruses in environmental samples or complex matrices. Although the target enriched panel applied in this study was designed in 2015, it favored the detection of Fur seal picorna-like virus which was discovered in 2017 (and since then never confirmed to occur in these animals), probably because of its similarity with other vertebrate picornaviruses. Here, we confirmed the presence of this virus in the 4 fecal pools studied and with high similarity, that may suggest a persistent infection in the animals studied. More studies with a broader range of samples should be conducted to confirm this hypotheses. Besides, variability observed among detected sequences suggests co-circulation of diverse fur picornavirus variants.

Other viruses never described before in this specie have been detected in this study: mamastrovirus, norovirus and adeno-associated viruses. None of the viruses were detected in all the 4 pools studied but some were present in 2/4 while others in 1/4 showing they are relatively prevalent in the fur seal population studied. Serum samples also showed no relevant sequences indicating that none of the viruses detected are causing acute infection or in case they cause, it happened without producing any important viremia in the studied animals or viremia was not simultaneous to fecal shedding. Actually, no evidence of disease was observed in any animal during handling, although no clinical evaluation was conducted.

Although specimens analyzed in this study were collected from the Antarctic region area with highest human presence, viruses detected are probably *A. gazella* viruses rather than viruses acquired directly or indirectly from humans. Further studies could be focused on a better characterization of detected viruses which would surely lead to the description of new *A. gazella* viruses.

Numerous invertebrate viruses have been detected, possibly as a result of the feeding habits of the animals studied. Most are undescribed viruses similar to other viruses that are likely to infect the same or similar invertebrate species, although crustacean viruses prevailed. This could represent an alternative approach for the study of animal diets especially when adequate monitoring and study of subject animals cannot be established.

The study of the viruses present in animal feces is a promising tool for the characterization of viruses affecting vulnerable species, viral discovery and as a tool for the study of dietary habits. Moreover, this approach can be conducted by studying groups of animals rather than individually and do not require animal capture or manipulation.

The data bank created in this study opens the chance of reanalyzing obtained results in the light of new descriptions of viruses circulating in Antarctica, an area which until now remains poorly investigated regarding viral occurrence.

## Methods

### Samples

**Figure 6:**
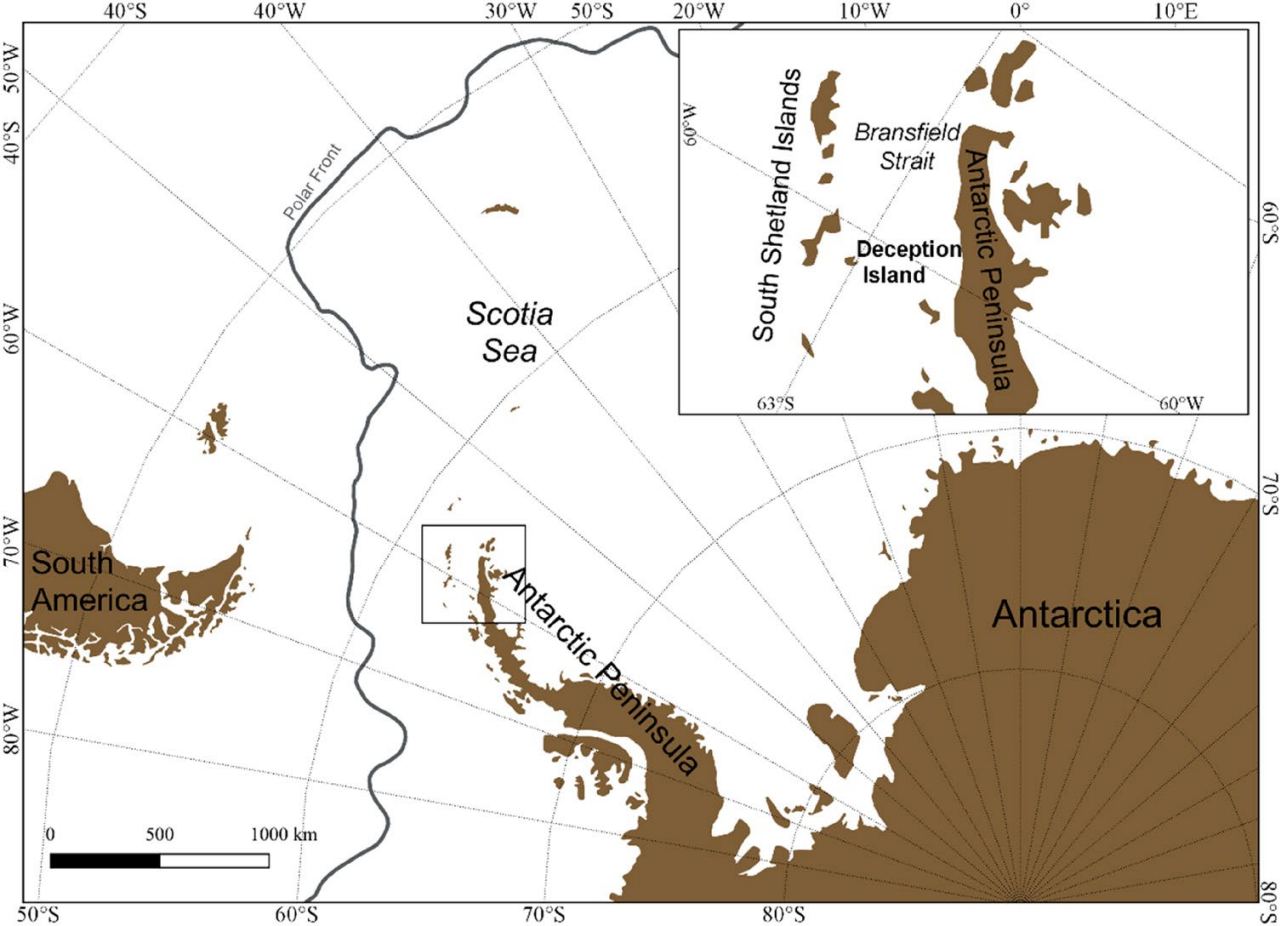
Map of the study area showing the Deception Island sampling location in the South Shetland Islands archipelago (Antarctic Peninsula). Map was created using the free software QGIS 3.10 (QGIS Development Team, 2018).

Sampling was conducted during late Antarctic summer (February–March 2019) at Deception Island (South Shetland Islands) (Fig. 6). Animal handling procedures were reviewed and approved by the Ethics Committee in Animal Experimentation of the University of Barcelona and the Government of Catalonia (project No 10292) in accordance with relevant guidelines and regulations. The procedures adhered to the ARRIVE guidelines and requirements of the ethics committee of the Spanish Polar Institute, who approved fieldwork under permit No CPE-2018-4.

Ten young (2–3 years old) and ten sub-adult (4–7 years old) male *A. gazella* were randomly captured, measured (standard length ranged from 127 to 173 cm), and classified according to body size and pelage coloration (Wilson and Mittermeier 2014). Age was estimated according to standard body length^56^. Most fur seals (n = 16) were captured using a hoop net and restrained, without sedative, in the same net. However, the largest individuals (n = 4) were chemically restrained with a combination of midazolam and butorphanol remotely administered using a dart (5 ml) shot by means of a CO_2_ Dan-Inject JM rifle (Børkop, Denmark)^57–59^. Once immobilized, about 2 ml of blood from the caudal digital vein was collected using butterfly blood-collection kits and vacuumed blood-collection tubes without any anticlotting factor. Blood samples were centrifuged in situ to separate serum and blood cells and serum samples were stored at −20 °C.

Fresh scats from young and sub-adult males were collected from the rookery at Collins Point with a small metallic shovel. Samples were wrapped in aluminium foil, stored individually inside zipped plastic bags at −20 °C. All samples were from non-individually identified young and subadult males.

### Viral concentration and nucleic acid extraction

Twelve fecal and 10 serum samples were selected for massive sequencing. Fecal samples were concentrated using an ultracentrifugation method as previously described^60^. Briefly, 1 gr of each fecal sample were incubated with 3.5 ml of glycine (0.25 N, pH = 9) on ice for 30 min, vortexing every 5 min. After the addition of cooled PBS 2×, the samples were centrifugated at 10,000 rpm at 4 °C for 15 min and the supernatants were then ultracentrifugated 1 h at 34,500 rpm and 4 °C.

Pellets obtained were resuspended in 100 µl of PBS and mixed in groups of 3, obtaining 4 pooled samples. These pooled viral concentrates were treated with TurboDNASe and subsequently total nucleic acid (NA) extraction was performed using the commercial QIAmp Viral RNA kit® (Qiagen Inc.) and the automated platform QIAcube. For the serum samples, NA from 10 individual specimens were directly extracted using the same extraction protocol.

### Library construction, probe-based enrichment and sequencing

NA were retrotranscribed using SuperScript IV (Life Technologies) and a random nonamer primer. With Sequenase 2.0 (Thermo Fisher Scientific), complementary cDNA strands were obtained and a PCR of 25 cycles was performed to obtain enough dsDNA quantity for libraries production. The resulting products were purified and concentrated with Zymo DNA Clean & Concentrate kit (Zymo Research) and quantified using Qubit 3.0 (Life Technologies).

Libraries for each sample were constructed in duplicate using KAPA HyperPlus Library Preparation Kit (KAPA Biosystems, Roche). Enzymatic fragmentation, end-repair, A-tailing reaction, and adapter’s ligation was performed. Each sample was indexed using the KAPA Dual-Indexed Adapter Kit (KAPA Biosystems, Roche). To select fragments between 250 and 450 bp, a post-ligation clean-up was followed with a double-sized size selection with the magnetic AMPure XP Beads (Beckman Coulter). Using a Ligation-mediated polymerase chain reaction (LM-PCR), the sample libraries were amplified and then purified.

The constructed libraries were pooled in duplicate and one of the replicates was hybridized with the VirCapSeq®-VERT Kit (Roche), a probe-based panel that covers the genomes of 207 viral taxa known to infect vertebrates^20^, at 47 °C for 20 h. Right after the hybridization, this captured multiplex DNA sample was recovered, cleaned-up and amplified using a LM-PCR. This post-capture PCR was afterwards purified, and the concentration and quality checked.

For each sample, captured and uncaptured libraries were sequenced with an Illumina Miseq® 2 × 300 bp platform. The negative control, from NA extraction, was incorporated in the sequencing run.

### Bioinformatics

Pair-end FASTAQ files generated from the sequencing were analysed using Genome Detective web-based software. Briefly, low-quality reads and adapters were trimmed using Trimmomatic, viral reads identified with DIAMOND alignment method, assembled with SPAdes and classified with a Blastx and Blastn against NCBI RefSeq viral database^61^. Viral assignations of the contigs obtained with this tool were reconfirmed by using BLASTn, to avoid possible miss-assignments derived from using a closed database.

The contigs generated were further analysed using Geneious R11. Alignments were generated by Muscle and phylogenetic trees were constructed using Geneious Tree Builder, constructed with the Tamura-Nei genetic distance model using the neighbour-joining method with 1000 bootstrap replicates with complete reference genomes and specific typing regions for Mamastrovirus.

## Data Availability

The datasets generated during the current study are available in zenodo under the DOI number 10.5281/zenodo.6517944.
